# Supplementary material for: Determining the effect of aging, recovery time, and post-stroke memantine treatment on delayed thalamic gliosis after cortical infarct
Source: Sci Rep. 2021 Jun 15;11:12613. doi: 10.1038/s41598-021-91998-3 (PMC8206333; doi:10.1038/s41598-021-91998-3)

Supplemental Figure 1.

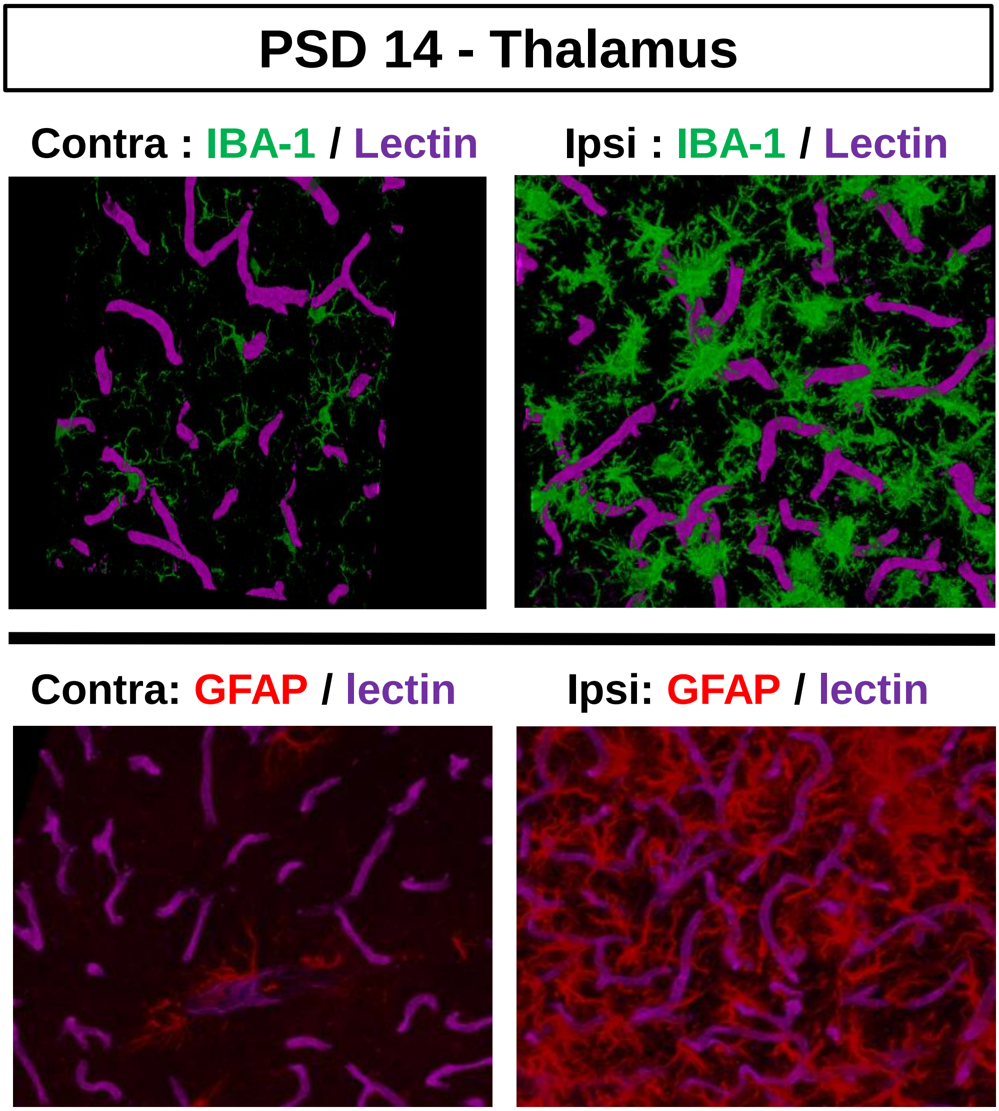

Supplemental Figure 2.

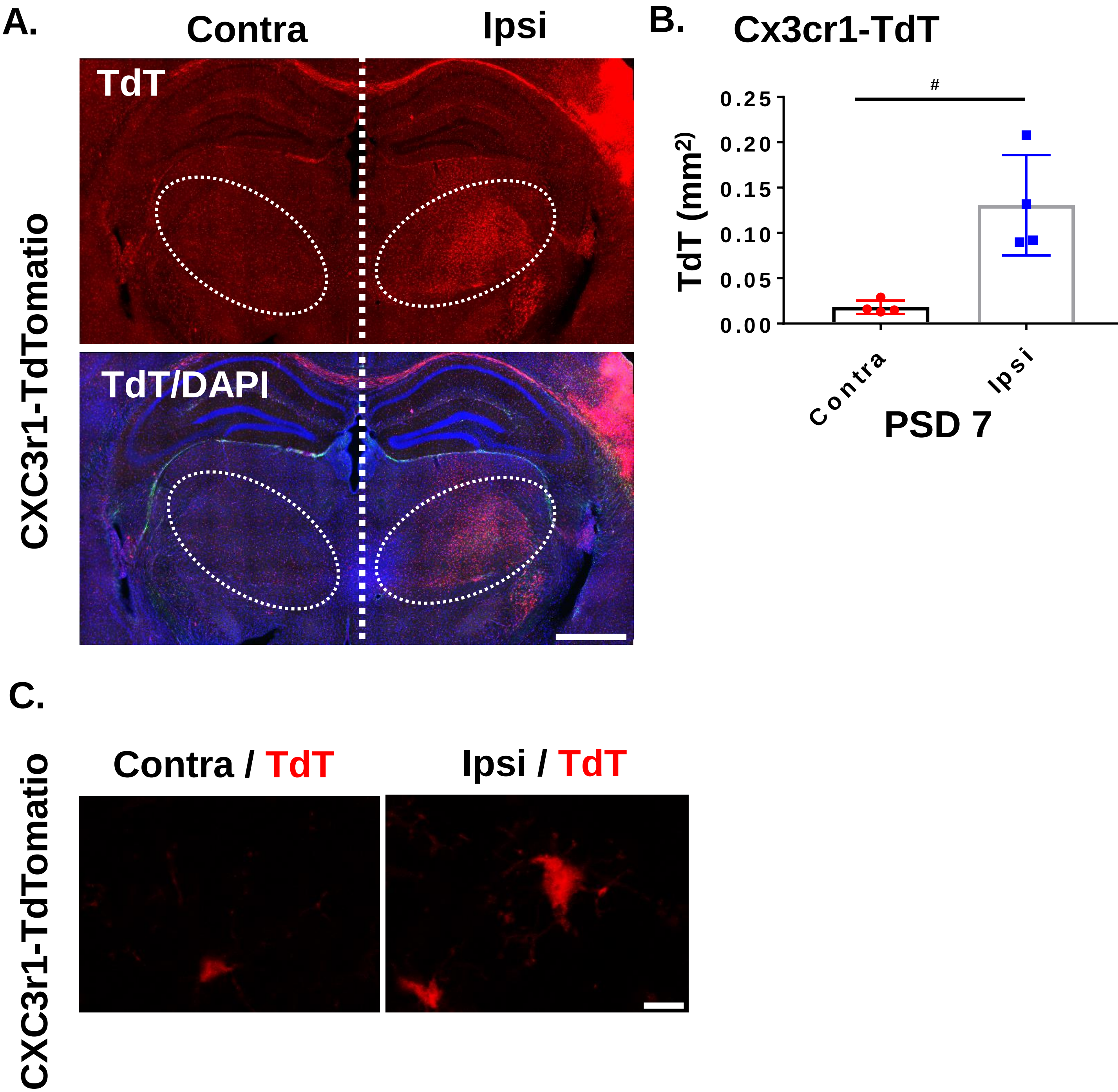

## Supplemental Figure 3.

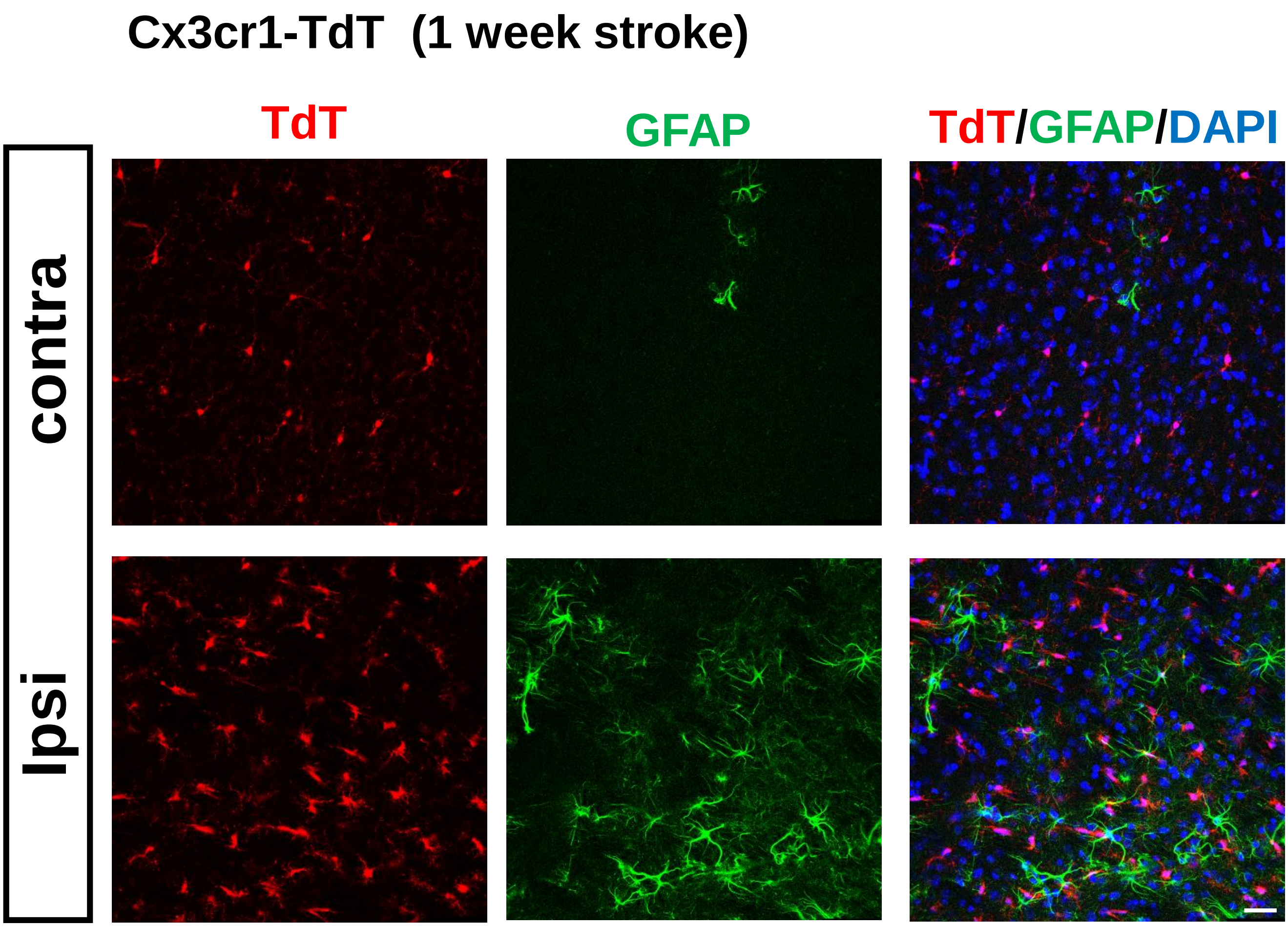

## Supplemental Figure 4.

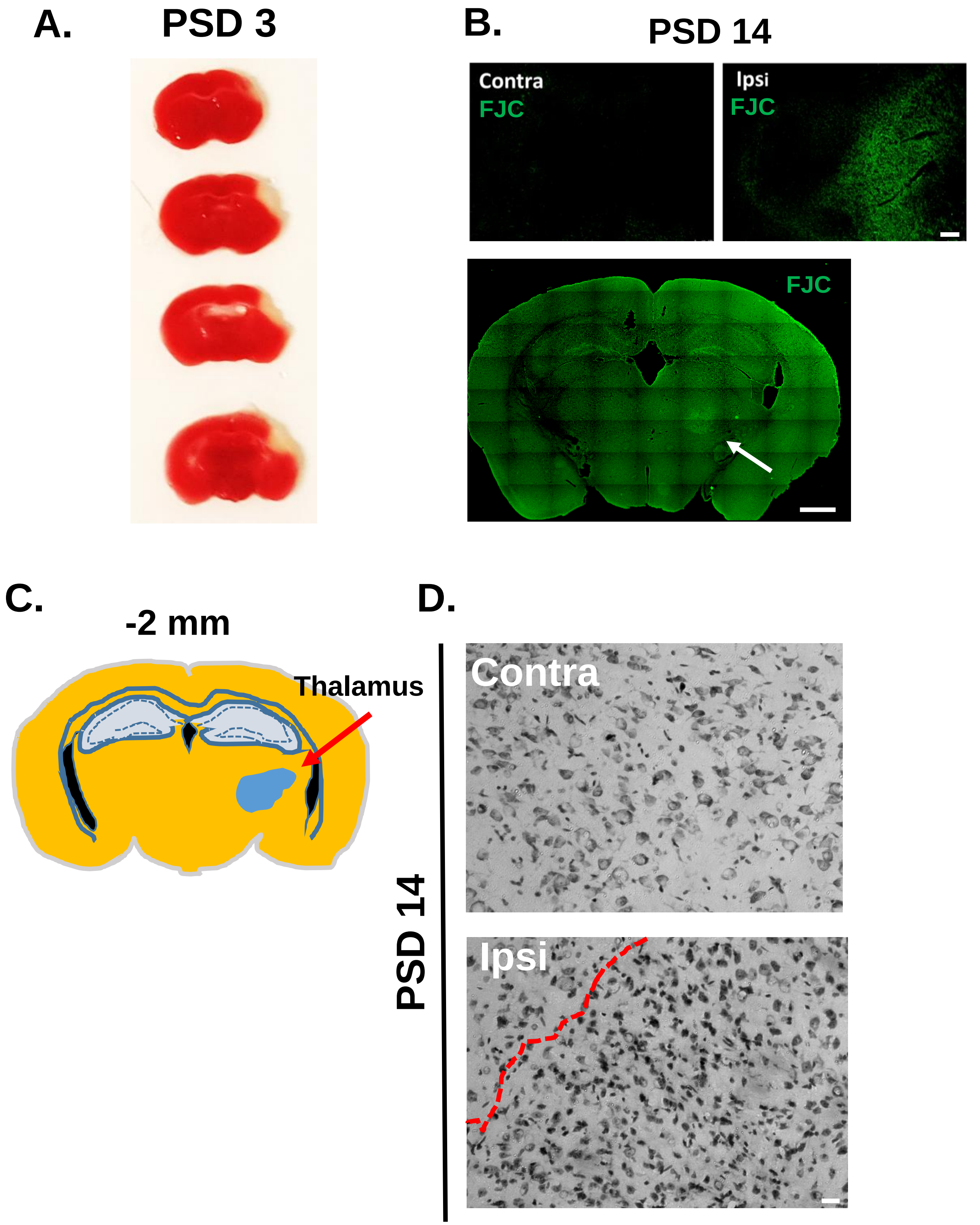

# Supplemental Figure 5.

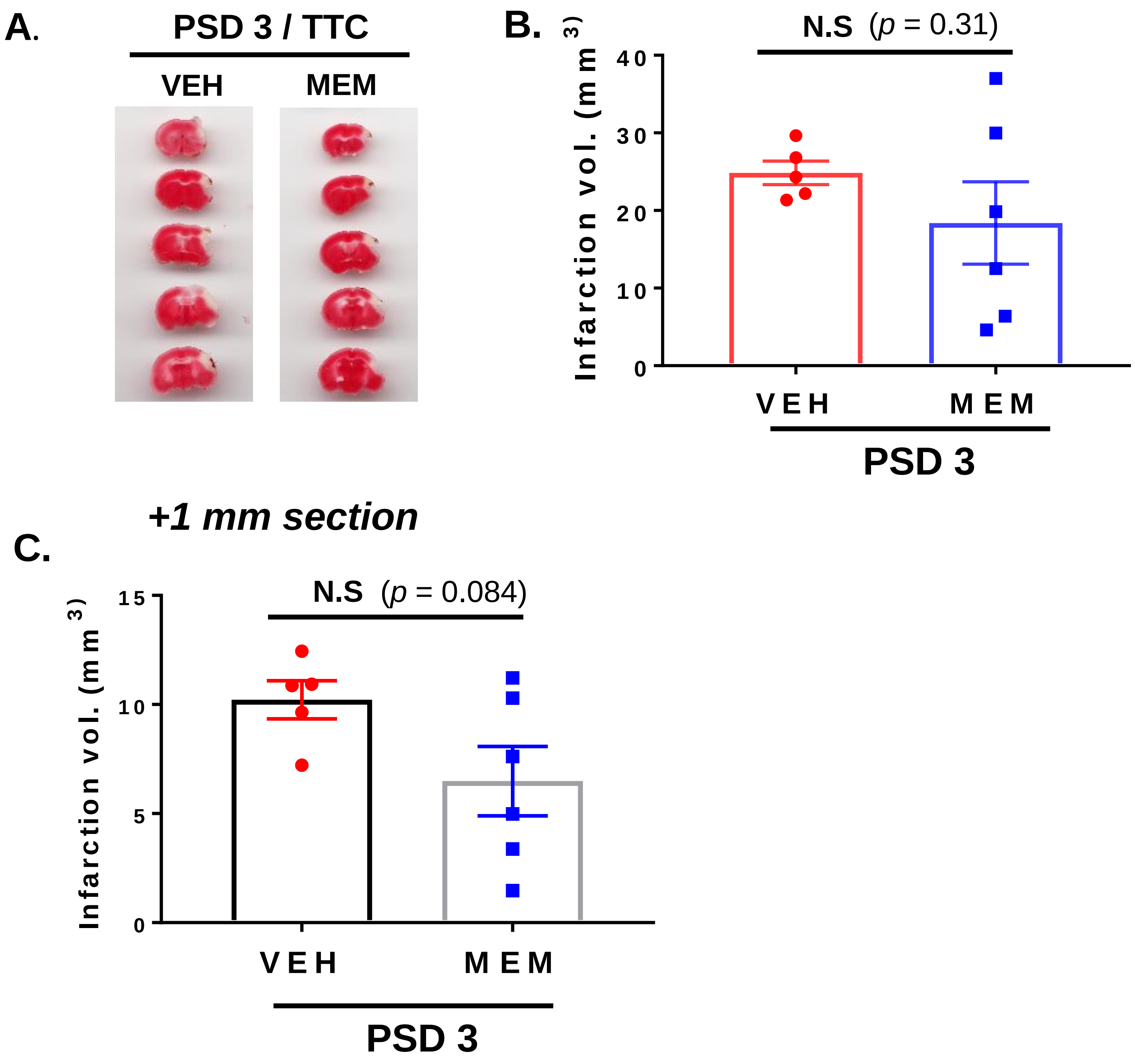

## Supplemental Figure 6.

A.

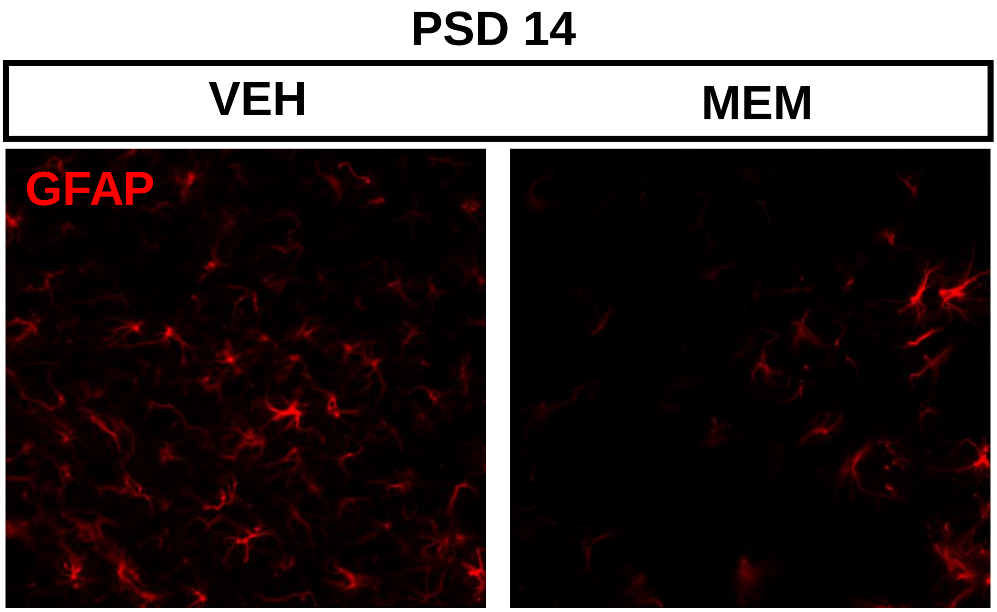


B.

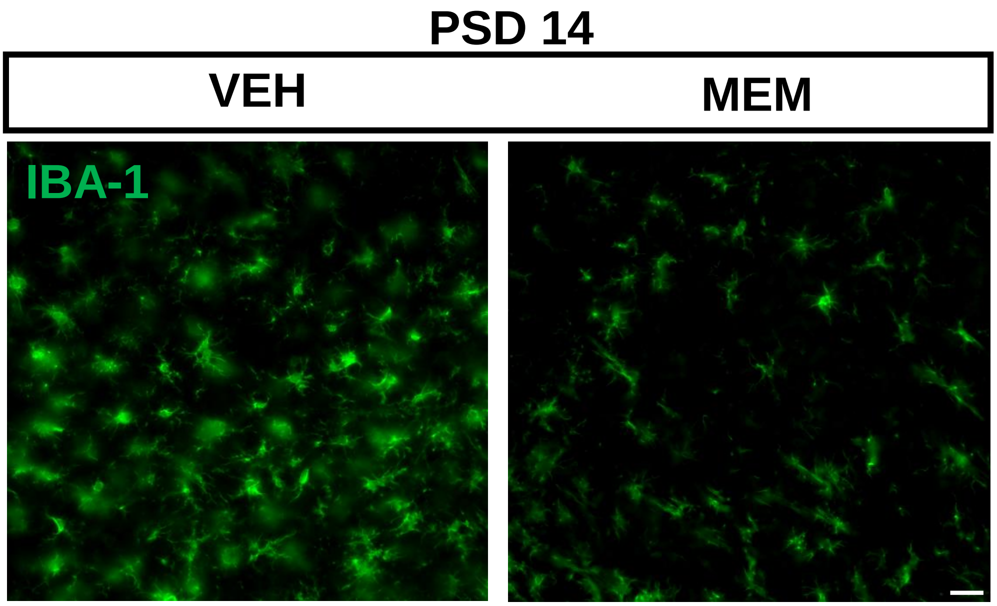

Supplement: Supplementary file 1 — Supplementary Information 1. [file 41598_2021_91998_MOESM1_ESM.pdf]
