# Supplementary material for: Determining the effect of aging, recovery time, and post-stroke memantine treatment on delayed thalamic gliosis after cortical infarct
Source: Sci Rep. 2021 Jun 15;11:12613. doi: 10.1038/s41598-021-91998-3 (PMC8206333; doi:10.1038/s41598-021-91998-3)
Supplement: Supplementary file 2 — Supplementary Information 2. [file 41598_2021_91998_MOESM2_ESM.docx]

**Supplemental Figure Legends**

Supplemental Figure 1. Activated glial cells in the ipsilateral thalamus show changes in morphology at PSD 14. 3D confocal reconstructed images (40X obj) showing enlarged, hypertrophic microglia (IBA-1- green, upper panel) and astrocytes (GFAP-red, lower panel). The activated glial cells closely associate with lectin stained microvessels (Lectin, Far-red).

Supplemental Figure 2. Cx3cr1-CreERT/TdTomato reporter mice demonstrate increased and hypertophied MG in the ipsilateral thalamus at PSD 7. The MG reporter mice underwent pdMCAO and TdTomato fluorescence was measured at PSD 7. TdTomato signal was significantly upregulated in the ipsilateral thalamus at PSD 7 (A, B, n=4, scale bar = 1 mm). C) 63X representative images show hypertrophic and activated microglia in the ipsilateral thalamus (scale bar = 10 µm). Nuclei were stained with DAPI (blue).

Supplemental Figure 3. TdTomato expression and GFAP expression (astrogliosis) were significantly increased at PSD 7 in the ipsilateral thalamus of Cx3cr1-CreERT/TdTomato reporter mice. TdTomato expression (red) and astrogliosis (GFAP, green) were significantly increased in the ipsilateral thalamus (n=4, 20X obj; scale bar = 20 µm). Nuclei were stained with DAPI (blue).

Supplemental Figure 4. Permanent distal MCAO induces primary cortical injury with secondary injury in the thalamus.

**A)** Representative TTC stained brain sections at PSD 3 showing primary injury largely restricted to the cortex. The pdMCAO was performed in young C57BL/6 mice. **B)** Fluoro-Jade C (FJC) staining showing degeneration of neurons in thalamus at PSD 14 (n = 5, scale bar = 100 µm in upper images, 1 mm in lower image). **C)** Illustration depicting the location of thalamic injury area from a brain section taken at -2 mm from bregma. **D)** Cresyl violet stained sections demonstrating degenerating neurons in ipsilateral, but not contralateral thalamus at PSD 14 (n = 5, scale bar = 20 µm). Degenerating neurons are evident by their dense pyknotic nuclei.

Supplemental Figure 5. Post-stroke treatment with memantine results in a non-significant reduction of total infarct volume and infarct at +1 mm from bregma at PSD 3. Representative images showing TTC staining **(A)** and infarct volume quantification (n=4-5, P=0.31) **(B)** for memantine and vehicle treated mice. **(C)** Primary infarct from +1 mm sections alone was also determined (n=5-6, P=0.084). Although both measurements showed a trend toward reduced cortical injury with memantine treatment, neither demonstrated statistical significance.

Supplemental Figure 6. Post-stroke treatment with memantine reduces astrogliosis **(A)** and microglial activation **(B)** in ipsilateral thalamus at PSD 14. Representative images showing effect of memantine treatment on astrogliosis and microgliosis in the thalamus (40X obj, scale bar = 20 µm).
